# 

**Published:** 1886-07

**Authors:** 


### CONTENTS.

ORIGINAL COMMUNICATIONS—Operation for Non-union of Fracture of Tibia, by J. M. Lewis, M. D., Mexia, Tex. 1
Placenta Prævia with Mal-presentation—Hysterotomy for Rigid Os Uteri, J. W. Hamilton, M. D. ........... 2
A Unique Case, W. H. Calfee, A. M., M. D., Waco... 6
Reminiscences of Prof. Gaillard; Substitute for Tracheotomy, W. N. Watt, M. D. ..................... 9
CORRESPONDENCE—Medical Legislation, M. M. Myers, M.D. 11
SOCIETY NOTES—Semi-annual meeting North Texas Medical Association .................................... 13
Rockwall and S. E. Collin Co. Med. Society ......... 15
Travis County Medical Society—"Germ Theory of Disease." ........................................ 15
Notice to Members P. M. B. A. of Texas .............. 16
A High Compliment to Dr. Q. C. Smith .............. 16
EDITORIAL—" Misguided Members of the Profession" and the Presidential Address .................................. 18
Death of Dr. William Jefferson Burt. Sec. T. S. M. A. 23
Do we need a Civil Service Reform in Medicine ? .... 25
Lawson Tait's Wonderful Success in Ovariatory ...... 27
"What are we here for ?" ............................ 29
"Colored Physicians" vs. "Negro Doctors." ........ 30
EDITORIAL NOTES.— ....................................... 37
ADVERTISERS' NOTICES.— ................................. 41

